# Pathogen detection in RNA-seq data with Pathonoia

**DOI:** 10.1186/s12859-023-05144-z

**Published:** 2023-02-17

**Authors:** Anna-Maria Liebhoff, Kevin Menden, Alena Laschtowitz, Andre Franke, Christoph Schramm, Stefan Bonn

**Affiliations:** 1 Institute for Medical Systems Biology, University Medical Center Hamburg-Eppendorf, Hamburg, Germany; 2 Department of Computer Science, Whiting School of Engineering, Johns Hopkins University, Baltimore, USA; 3 Department of Genome Biology of Neurodegenerative Diseases, DZNE, Tübingen, Germany; 4 I. Department of Medicine, University Medical Centre Hamburg-Eppendorf, Hamburg, Germany; 5 Institute of Clinical Molecular Biology, Christian-Albrechts-University of Kiel, Kiel, Germany; 6 Martin Zeitz Center for Rare Diseases, University Medical Center Hamburg-Eppendorf, Hamburg, Germany; 7 Hamburg Center for Translational Immunology (HCTI), University Medical Center Hamburg-Eppendorf, Hamburg, Germany

**Keywords:** Metagenomics, Pathogen detection, RNA sequencing

## Abstract

**Background:**

Bacterial and viral infections may cause or exacerbate various human diseases and to detect microbes in tissue, one method of choice is RNA sequencing. The detection of specific microbes using RNA sequencing offers good sensitivity and specificity, but untargeted approaches suffer from high false positive rates and a lack of sensitivity for lowly abundant organisms.

**Results:**

We introduce Pathonoia, an algorithm that detects viruses and bacteria in RNA sequencing data with high precision and recall. Pathonoia first applies an established k-mer based method for species identification and then aggregates this evidence over all reads in a sample. In addition, we provide an easy-to-use analysis framework that highlights potential microbe-host interactions by correlating the microbial to the host gene expression. Pathonoia outperforms state-of-the-art methods in microbial detection specificity, both on in silico and real datasets.

**Conclusion:**

Two case studies in human liver and brain show how Pathonoia can support novel hypotheses on microbial infection exacerbating disease. The Python package for Pathonoia sample analysis and a guided analysis Jupyter notebook for bulk RNAseq datasets are available on GitHub.

**Supplementary Information:**

The online version contains supplementary material available at 10.1186/s12859-023-05144-z.

## Background

A common approach to obtain insights into molecular mechanisms of disease is to sequence and analyze patients’ transcriptomes and compare them to transcriptomes of healthy controls. These experiments capture gene expression changes in human cells that might underly the disease process, but they can also capture transcripts of viruses or bacteria that infected those cells. In many cases, transcript information that cannot be aligned to the human genome or transcriptome is discarded as unspecific or contaminant. However, these non-human transcripts might be of microbial origin and provide important insights into disease.

Especially in recent years it has become clear that the human body harbors a vast amount of non-human cells. Certainly, these cells, mostly bacteria, are predominantly found in the gut and skin microbiomes, but other human tissues show abundance of microorganisms as well. For example, the healthy human blood microbiome is discussed by Castillo et al. [1] and shown by Martí [2]. Even the healthy brain is suspected to contain bacteria permanently according to Roberts et al. [3] although there is disagreement in the community [4].

For understanding the effect of certain agents in human tissues, dual RNA-sequencing experiments [5] can be conducted in vitro. Nevertheless, it might be unknown that a pathogen relates to a disease in the first place and for discovering it and its co-morbidity in a disease state, samples and data from patients are needed.

The idea of finding foreign RNA in patient’s sequencing data has been proposed before, for example by Sangiovanni et al. [6] and Rahman et al. [7]. The non-human part of a sample can be analyzed as a metagenome. Many publicly available datasets have been analyzed with this notion by Simon et al. [8] who created a database for a wide search of potential disease related pathogens. Similarly, we created an online database of re-analyzed public small RNA experiments [9], that contains a wide range of diseases and pathogens.

Metagenomes, as known from the studies of microbiomes and environment, e.g. soil, have their own challenges [10] but many tools exist to measure their abundance of bacteria and viruses, as proposed by Wood et al. [11], Kim et al. [12] and Alawi et al. [13]. The latter propose DAMIAN in which sequencing reads are assembled before aligning to a pathogen database which results in high confidence in the analysis outcome. However, important evidence might be disregarded when only little genetic material is available. The first two tools, Kraken 2 and Centrifuge on the other hand classify reads based on subsequences of reads that match organisms in their corresponding index. Therefore, they can pick up even small evidence for pathogenic presence.

Nevertheless, the non-human part of a transcriptomic sample is noisy, which means that it contains majorly sequencing reads which do not have a bio-medical background. These reads may instead stem from (human) processing contamination, poor sequencing quality and intentionally added sequences as part of the experimental protocol, e.g. primers. Falsely detected organisms (false positives) are a known issue for metagenomic data analysis, as discussed by McIntyre et al. [14]. However, the problem is even bigger in the metagenomic samples, which we observe as a side-effect of RNA-seq.

Targeting this issue, Recentrifuge [2] was developed for distinguishing the real signal from this noise by comparing all samples in a dataset of metagenomic abundance data. Most metagenomic analysis tools measure species’ abundance by classifying for each sequencing read the organism it may stem from and summing up the reads according to their taxonomy. Analogous to the gene-count matrix, an organism count matrix is created. The problem in this approach is the handling of sequences that have many copies in the sample due to some processing steps. They are often aligning to a random organism, which is then showing high abundance because of an exceedingly repeated read. In contrast, the traces of real pathogens may have a very low abundance and would drop out of the analysis. Furthermore, chimera, which are combined sequences of different species, cannot be correctly identified [15].

Therefore, we propose a solution which is considering the sample as a whole and measuring abundance of microorganisms across sequencing reads. Retaining an improved measure of abundance, common sequencing contamination can be differentiated from biological effects through the group-wise comparisons of their mean abundance.

## Results

Here, we are describing the Pathonoia algorithm, trying to overcome the problem of falsely detected organisms (false positives (FP)) in a metagenomic sample. Our motivation originates in samples which contain a low number of true positives (TP) combined with low-quality RNA sequences or sequences that got artificially enriched when processing the sample. For these metagenomic samples, which are often a side product of transcriptome sequencing of a host, it is challenging to gain high specificity for the detected organisms.

### The Pathonoia algorithm

**Fig. 1:**
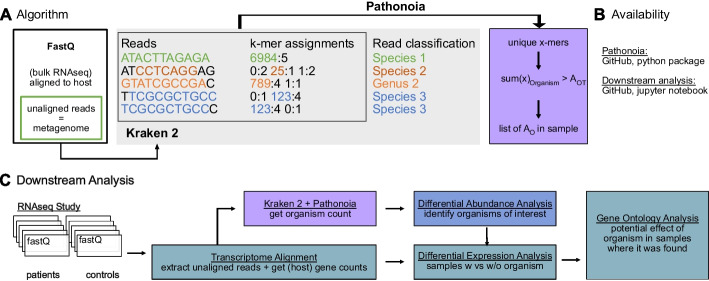
Pathonoia toolkit. **A** The algorithm analyzes unaligned RNA-seq reads, based on Kraken 2. Kraken generates k-mer assignments and a taxonomic classification for each read (grey box). Pathonoia uses all k-mer assignments of a sample and combines them into a non-read-count based abundance metric $A_{O}$. **B** Pathonoia and the downstream analysis template are available on GitHub. **C** The analysis workflow for a dataset. A transcriptome alignment yields gene counts and unaligned reads which are analyzed by Pathonoia (A). A differential abundance analysis reports organisms that are more frequent in one sample group compared to another (examples in Fig. 3B, F). An “organism of interest” (OoI) can be selected for understanding its role in a sample group. Samples with ($A_{OoI}>0$) and without ($A_{OoI}=0$) the OoI are compared in a differential gene expression analysis using the gene counts. A gene set enrichment analysis of de-regulated genes may uncover the pathways affected by the OoI

Currently, Kraken 2’s [11] metagenomic alignment is widely used due to its excellent speed and accuracy [14, 16]. Our proposal of Pathonoia is using Kraken 2’s k-mer matches to all existing bacterial and viral genomes in the NCBI database and replace its read classification step with a sample wide evaluation for organism abundance. Figure 1A shows an overview of the algorithm. The abundance metric $A_O$ refers to the summed length of unique subsequences of an organism. A detailed description is given in the methods section. The code and Python package is available on GitHub (Fig. 1B). Furthermore, we propose a downstream analysis for gaining biological results on a dataset, using Pathonoia for measuring abundance in each sample (Fig. 1C). Two case studies are described below in the section “Downstream analysis framework”.

**Fig. 2:**
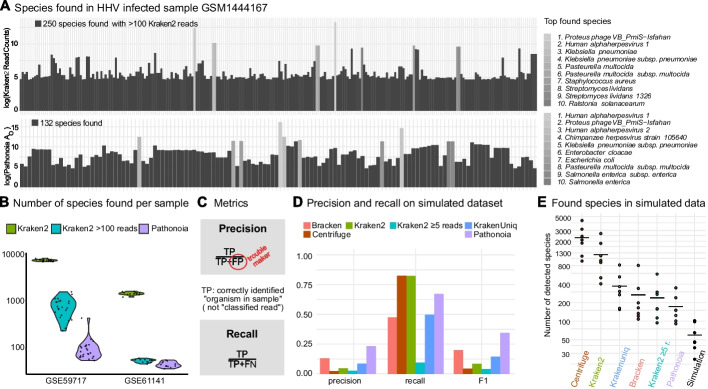
Pathonoia reduces number of false positives (FP) in noisy metagenomic samples. **A** The spectrum of species is shown, as reported by Kraken 2 and Pathonoia for a cell line sample infected with* Human Herpes Virus* (HHV). The top 10 most abundant species are highlighted. Kraken 2 reported 7262 organisms of which 250 are shown that have $>100$ reads. Pathonoia lists 132 organisms and Herpes viruses ascend in the ranking of reported species. **B** Number of reported species in two datasets (12 and 24 samples) by Kraken 2, Pathonoia and Kraken with threshold (organism *detected* if $>100$ reads counted). A lower number of detected organisms is desirable since it reduces the number of FP. **C** Pathonoia aims to improve the precision of detected organisms in a sample. FP (sequencing errors, other sample bias or random alignments, especially with poor quality reads) should be removed. **D** Average precision, recall and F1 for a simulated dataset, evaluated for Kraken 2-based algorithms and Centrifuge. Recall is the highest in Kraken 2 and Centrifuge. With removing FP from the Kraken results, every algorithm also loses some TP (recall goes down). **E** Number of species detected in simulated dataset. High recall in D is explainable by the high number of species that each algorithm finds

**Fig. 3:**
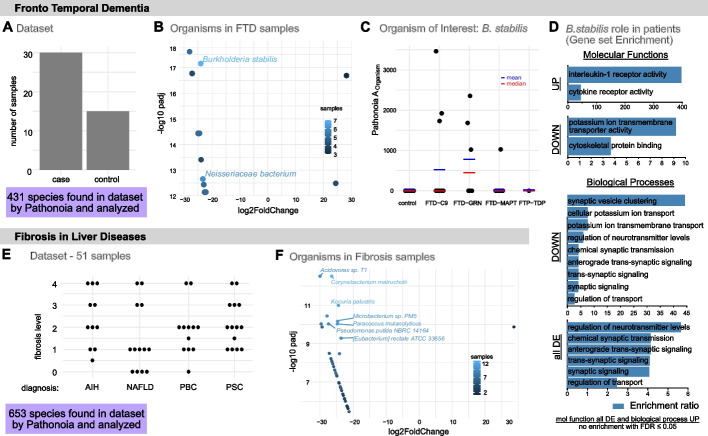
Case studies: analyzing datasets with Pathonoia. **A**–**D** Fronto Temporal Dementia. **A** The dataset contains 30 cases of FTD (sub-groups shown in C) and 15 controls. Pathonoia reported 431 organisms over all samples. **B** The volcano plot shows 12 differentially abundant organisms, ten of them up-regulated in FTD samples. The color scale shows the number of samples containing the organism. **C** *B. stabilis* was chosen as OoI. $A_O$ is given across samples. **D** Three gene sets from a differential expression analysis between patients with and without *B. stabilis* (34 up-regulated genes, 109 down-regulated genes, in total 143) were compared in an over-representation analysis with gene sets related to Molecular Functions and Biological Processes. (By *B. stabilis*) up-regulated genes hint towards an immune reaction in the FTD patients. The Biological Processes relate to neural pathways. **E**–**F** Fibrosis in Liver Diseases. **E** A dataset with 51 human liver samples from patients with different liver diseases and fibrosis levels comprises 653 reported species by Pathonoia. **F** A differential abundance analysis of samples with and without fibrosis lead to 41 organisms of which only one was up-regulated in two non-fibrotic samples. Seven organisms were present in more than nine fibrotic samples

### Improved abundance profile per sample

The evaluation of a biological sample with Kraken 2-only and Pathonoia is visualized in Fig. 2A. Pathonoia is able to reduce the number of (falsely) detected organisms, but also improves the profile of possibly abundant organisms, i.e., the differences in the abundance metric are larger for the included species. The sample in Fig. 2A is a *Human herpes virus*-infected in vitro sample [17]. In vitro samples should be comparably clean, i.e., contain fewer contaminating organisms. However, Kraken 2 identified 250 species with over 100 reads in the non-human reads of the sample, while Pathonoia reduced this number to 132. Pathonoia detected three different herpes virus strains in the top hits, while Kraken 2 only detected the major one, *Human alpha herpes virus 1*. Furthermore, *Proteus phage VB PmiS-Isfahan* was the top hit for the Kraken 2 algorithm. Its genome is relatively short (3.8 MB), and it was not mentioned to be part of the sample. As an unexpected contaminant, it might be a false positive which Pathonoia ranked lower. *Klebsiella Pneumonaie* and *Pasteurella multocida* were found through both algorithms with relatively high abundance and are species who commonly appear in our environment [18, 19].

### Reduced number of detected species

Figure 2B shows the reduction of detected species in samples of two in vitro datasets [17, 20] between Pathonoia, Kraken 2 and a version of Kraken 2 in which only species are taken into account that had more than 100 reads assigned. The Kraken 2 filter (${>100}$ reads) reduces the number of detected species. For a simulated dataset, containing twelve to 50 organisms per sample, we evaluated several tools reflecting the same reduction of detected species in Pathonoia over Kraken 2 and Centrifuge [21] (Fig. 2E). We observe that other algorithms also reduce the number of detected species significantly, such as KrakenUniq [22], Bracken [23] and Kraken 2’s filtered version. However, Pathonoia’s mechanism to only account for unique sequences within a sample, decreases the number of species while also decreasing the ratio of FP (Fig. 2C) effectively, as shown in the following section.

### Improved precision and F1 score in simulated dataset

Contaminating sequences that originate from sample processing steps tend to result in many identical reads. These sequences may not be present in the Kraken 2 index of bacteria and viruses, but their k-mers can match organisms in the database by chance. This leads to a false classification of the read (by Kraken 2) and with many copies present, this false positive gains a high read count (abundance metric). Pathonoia does not evaluate individual reads or take their quantity into account. It intentionally excludes identical sequences from the counting because a higher abundance of a natural organism would result in more diverse sequences in the sample. Furthermore, Pathonoia rewards longer matches to an organism’s genome than shorter ones (compare Methods and Additional file 1: Fig. SF1).

As displayed in Fig. 2D, with this approach Pathonoia is achieving greater precision on the detected organisms than Kraken 2’s read based evaluation alone and other Kraken 2-based abundance measuring techniques, like the filtered Kraken 2 approach ($\ge 5$ reads), Bracken and KrakenUniq. We further evaluated Centrifuge for comparison to Kraken 2-based techniques and found that it detects even more FP, resulting in the lowest precision close to filtered Kraken 2. We measured this precision on an artificial dataset (mentioned above), containing seven samples with organisms found in common human microbiomes or environmental metagenomes, for having a ground truth of organisms present, and especially not present in the samples.

Comparing Fig. 2D and E, it can be noted that the high number of detected species in a sample result in a high recall for Kraken 2 and Centrifuge since the recall metric does not consider FP (Fig. 2C).

Furthermore, Pathonoia achieves the highest F1 score, which is the harmonic mean of precision and recall, as compared to the other abundance measuring techniques. Overall, Pathonoia has an F1 score of 0.34 and the pure Kraken 2 algorithm has 0.1, yielding an improvement $0.343/0.085 \approx 404\%$.

The downstream analysis (Fig. 3) helps to put samples and abundance measures in context of a dataset and can remove further sample bias.

### Downstream analysis framework

Making use of Pathonoia’s abundance measures of species, we propose a downstream analysis for gaining biological results on a dataset (Fig. 1C). Here, we propose to compare case and control samples in a differential abundance analysis to uncover the potential involvement of a species in a condition. If the metagenomic sample originates from a transcriptome sequencing experiment, we propose to analyze samples with and without pathogen presence in a differential gene expression analysis, followed by a Gene Ontology analysis, based on differentially expressed gene sets, with WebGestalt [24] (see Methods). We provide a generic Jupyter Notebook to execute this analysis on GitHub (Fig. 1B).

### Case study: frontotemporal dementia (FTD)

Figure 3A–D show the downstream analysis results for a dataset [25] containing 30 cases of FTD and 15 controls (Fig. 3A). Pathonoia reported 431 organisms over all samples, of which 12 are significantly (p-adj. $<0.05$) differentially abundant, ten of them up-regulated in FTD samples (Fig. 3B). *Burkholderia stabilis* appears in the highest number of samples as compared to other organisms (7 cases, Additional file 1: Fig. SF8) and is therefore chosen as organism of interest (OoI) for further analysis. Figure 3C shows its abundance in Pathonoia’s metric $A_O$ in the different sample groups, stratified by disease sub-type. A differential expression analysis between FTD patients with and without *B. stabilis* lead to a gene set of 34 up-regulated and 109 down-regulated genes, in total 143. These three gene sets were compared with gene sets describing Molecular Functions and Biological Processes in a Gene Ontology analysis. Figure 3D shows the pathways’ significant (FDR$\le 0.05$) enrichment ratios for immunological and neurological pathways.

### Case study: fibrosis in liver diseases

Figure 3E–F show the downstream analysis results for an in-house dataset containing 11 samples from patients with Autoimmune Hepatites (AIH), 13 from Non-Alcoholic Fatty Liver Disease (NAFLD), 12 from Primary Biliary Cholangitis (PBC) and 14 from Primary Sclerosing Cholangitis (PSC) with different levels of fibrosis (Fig. 3F). 653 species were reported by Pathonoia of which 40 were significantly (p-adj. $<0.05$) up regulated in fibrotic samples over non-fibrotic ones.

## Discussion

In an RNA-seq sample, a fraction of sequencing reads does not align to the organism’s transcriptome that is being studied. Pathonoia was developed for making use of this situation and detecting additional organisms in this data. With our method, further information can be found about the biological sample, as well as evidence for potential infections in the host, e.g., human tissue.

### Algorithm performance

Kraken 2 and Kraken 2-based algorithms measure abundance of organisms based on read counts. Previously, these algorithms were adopted for the above mentioned task. We compared our algorithm against these commonly used Kraken 2-based abundance measuring techniques. Pathonoia’s precision exceeds theirs and showed a reduction of false positives.

We used a simulated dataset for benchmarking of Kraken 2-based abundance measuring techniques. For all samples, the recall is significantly higher than precision because of the high number of organisms found with Kraken 2. Most TP are discovered resulting in a high recall, but also a high number of FP are detected, which in turn reduces precision (Fig. 2C). For improving certainty about Kraken 2-detected organisms, a cut-off can be introduced (“Kraken 2 min five reads” in Fig. 2D) for reducing random hits. As a result, also TP are reduced and both, recall and precision are dropping. Looking at alternative abundance calculations with Bracken and KrakenUniq, they do perform better than Kraken 2 and are closer to Pathonoia’s values.

The performance difference between KrakenUniq and Pathonoia is surprising since their approaches are similar. They differ in two small aspects. First, Pathonoia gives a higher score when several k-mers of the same species appear in a row (x-mers), which makes it less dependent on k. Second, KrakenUniq is estimating the cardinality of distinct k-mers using the HyperLogLog algorithm while Pathonoia stores x-mers in a hashmap.

As Kraken 2 is the base for all benchmarked algorithms except Centrifuge, organisms can only be detected if they were detected by Kraken 2 (with some exceptions, see Additional file 1: Sect. S1.1). With removing FP from the Kraken 2 results, every algorithm also loses some TP, which is why the recall goes down. Thresholding alone worsens precision. For precision and the balanced F1 score, Pathonoia achieves best results.

During Pathonoia's algorithm design phase, we considered that larger genomes naturally “attract” more random matches. Hence, we evaluated the effect of a normalization of $A_O$ regarding the organism's genome length, using publicly available in vitro samples (no figure). However, this biased the results towards shorter genome sizes, because the genome lengths vary much more between species than the collected evidence $A_O$. This is due to the type of samples that we observe. They contain only few traces of an infection and a coverage measure would not be beneficial for detecting these traces.

For the actual read-classification task, which Kraken 2 and other tools were built for, precision and other performance measures are usually over $95\%$ [16]. We showed, that with measuring performance based on the finding of specific organisms instead of correct classification of reads, the F1 score drops to less than $20\%$ with common techniques. Pathonoia, in comparison, reaches up to $30\%$, which is much better but still not practical if used as stand-alone solution. We hope to start a new direction for the development of tools for gaining results with better biological interpretability. Pathonoia shows the best performance for the task of organism detection. With it we suggest a methodology, that is based on measuring abundance on sample level through adding up distinct subsequences of nucleotides for the aim of specific indication of abundant organisms in a noisy metagenomic sample.

### Real-world application

When looking at non-simulated sequencing data, we observe various artifacts (Fig. 2A). Some organisms seem to be present in the sample even though their presence was not mentioned or explained in the original study. They can be detected with both Pathonoia and Kraken 2. The most questionable one is *Proteus phage VB PmiS-Isfahan*. Phages are frequently used in bio-technical applications [26] and may serve for a quality check for the sequencing data. However, we could not find evidence for this hypothesis. Further, *Klebsiella Pneumonaie* and *Pasteurella multocida* were found with relatively high abundance and are species that commonly appear in our environment [18, 19]. They may have entered the sample during its collection, transport or manual processing. *Salmonella enterica* and *Staphylococcus aureus* on the other hand are extensively researched and sequenced organisms which inhabit and infect the human body [27, 28]. Nonetheless, it cannot be determined which of these organisms was actually in the sample or contaminated it during the handling of a human being, for example in a hospital setting. Since this sample was infected in vitro, we expect it to be comparably “clean”. Pathonoia could detect the known HHV infection, but also reduced the noise of random computational hits.

Another artifact in biological data may be chimera or cross-species reads. As Pathonoia collects evidence for the presence of organisms across reads, the evidence for two different organisms can potentially stem from the same read. However, partial reads alone would not pass the $A_{OT}$ threshold which assures stability in the algorithm.

### Downstream analysis

For increasing certainty about the detected organisms, samples should be considered in the reference of a whole dataset. Factors like sampling bias and sequencing noise may shadow the observations made on a single sample. We propose a downstream analysis comparing sample groups of a dataset to reduce the amount of sequencing artifacts and only find organisms which are unique to a defined condition. Furthermore, we suggest analyzing the transcriptome data of the host, from which our data was derived, in the light of the results of that comparison. Comparing the transcriptome of samples containing a bacterium versus samples not containing it within the same condition can give important indications on which effect the bacteria can have on the host.

Pathonoia and the downstream analysis are provided online on Github: https://github.com/kepsi/Pathonoia. It produces several plots for an exploratory analysis. Some decisions must be taken manually, as for example the selection of an organism of interest, once displayed with their differential abundance. Analyzing the results carefully is important, since a group difference in pathogenic load can come as well from a badly designed or executed experiment. Yet, to understand if the organism of interest has a biological origin, the transcriptome data can be used.

### Evidence for novel hypotheses

An FTD case study indicates that some patients may have had a *B. stabilis* infection in the brain, as by *B. stabilis* up-regulated genes in FTD patients are enriched in immune reaction related pathways and the enriched Biological Processes relate to neural activity. It was shown in literature that members of the *Burkholderia cepacia complex*, to which *B. stabilis* belongs, were able to infect human brain tissue and cause brain abscesses [29] and meningitis [30]. Furthermore, *Burkholderia pseudomallei*, belonging to the same genus, are up-regulated in two independent, publicly available datasets concerning neurodegenerative diseases as shown in the Small RNA Expression Atlas [9] platform (Additional file 1: Table ST4). Nonetheless, it remains unclear whether *B. stabilis* plays an important role in FTD or could enter the brain for example due to already degenerated brain tissue. Outbreaks of *B. stabilis* in hospitals are reported frequently [31, 32]. There was a major outbreak in Swiss hospitals due to contaminated washing gloves in 2016 [33]. It is not known if patients from this cohort were treated there or in another hospital which had an unreported outbreak and if they could have been infected there. Further experiments should be conducted, such as dual RNA-seq experiments [5], which may help to answer, for example, the question how brain cells get affected by *B. stabilis*’ presence, and if it may be able to cause disease or change its progression.

Fibrotic livers are prone to bacterial infection through translocation from the gut [34] and we could observe this in our samples as well. We found 41 species significantly differentially abundant where only one of them was highly abundant in 2 non-fibrotic samples. For the three most significant organisms, we conducted the human transcriptome differential expression analysis between fibrotic samples with and without the species, but it did not show any differentially expressed genes (for two species, *Acidovorax* sp.T1 and *Kocuria palustris*) and no enriched pathways for 19 differentially expressed genes of the third organism (*Corynebacterium matruchotii*). This may support the hypothesis, that liver fibrosis is not a reaction to infection, but rather allows infection to happen and that healthy tissue is less prone to host bacteria.

## Conclusion

Our aim was to make use of the non-host part of RNA sequencing experiments and find potential infections or microbial abundance in the tissues under study. It is the nature of lowly abundant organisms that any algorithm cannot detect them with high certainty. Many random hits lead to noise in the data. With our proposed algorithm Pathonoia, it is possible to polarize some organisms from the noise. In contrast to the aim of other metagenomic algorithms, we focus on the detection of organisms, i.e., answering the question if an organism is present in the sample at all. Also, we wanted to overcome the commonly high false positive rate, which is especially increased in the kind of data we are focusing on. By considering the full sample instead of individual sequencing reads, we reach $400\%$ improvement in precision and uncovered pathogenic traces from noisy data.

Furthermore, we proposed a downstream analysis for detecting microbiotic abundance in a group of samples within a dataset and for suggesting their influence on the host’s transcriptome. Two case studies give examples of the added value of our algorithm Pathonoia. They show that the developed algorithm can model biological context and may be able to support building new hypotheses and getting insights to disease.

## Methods

First, we describe Pathonoia, which has the goal to uncover organic RNA-sequences from metagenomic sequencing reads of unknown origin. These reads may stem from the unaligned portion of RNA sequencing data from, e.g. human, transcriptome samples. Additionally, we describe our benchmarking methods.

Second, we describe an analysis pipeline based on the output of Pathonoia. Here, the goal is to distinguish biological signals from experimental contamination. We provide two exemplary studies. This pipeline is available as a guided analysis template in a Jupyter Notebook on GitHub.

### Pathonoia

Non-host-mapping reads are assumed as input for Pathonoia for detecting biological signal in noisy RNA-sequencing data. SAMtools [35] can be used to assemble the corresponding fastQ files after using any aligner (see Additional file 1: Sect. S1 for further details). This input is processed in two major steps: metagenomic alignment with Kraken 2 [11] and Pathonoia’s sample-wide aggregation of k-mers.

Kraken identifies the lowest common ancestor (LCA) for each k-mer in a sample. In Kraken 2, the developers optimized the index size and classification speed, using a minimizer scheme. A minimizer is a subsequence of a k-mer with length *l*. It can be used as a representative of the k-mer at the cost of classification accuracy. We use minimizer and k-mer lengths $l=k=31$ for highest precision settings of Kraken 2 and the Kraken 2 index for all viral and bacterial genomes in the NCBI nt database. Precision is lower for minimizers *l* with $l<k$ as there could be two k-mers from different species with the same minimizer. Pathonoia cannot work correctly with Kraken indexes that use $l<k$. The LCA for a k-mer is the most specific taxonomic description of a set of organisms that share this sequence. We use the taxonomic identifier (taxID) for the implementation of the algorithm. The outcome of the alignment step is the *kraken-align* file, which contains the classified k-mers for each read of a sample. One example format of a classified read is:

C  K025:418/1  Shamonda o.virus (taxid 15915)  100 0:20 15915:7 0:15 15915:6 1:5 0:2 230658:1 0:10

The five fields are:

1. *C* or *U* if the read was classified or not.
2. The read identifier as given in the fastQ file.
3. The taxonomic name and taxID with which it was classified according to the Kraken algorithm.
4. The original read length.
5. Sequence of pairs in format taxID:Y, where Y is the number of k-mers in a row which are classified with the same taxonomic identifier.

In this example, 20 k-mers could not be identified (taxID = 0), seven k-mers belong to the *Shamonda orthobunya virus* genome, followed by 15 unidentified k-mers and so on. When adding up the number of k-mers in a read, in this example $20+7+15+6+5+2+1+10 = 66$, the result is always the read length subtracted by $k+1$, here $100-(31+1) = 66$.

The second and key step of Pathonoia is the interpretation of the Kraken alignment output and detaching it from the notion of reads. It is also described in Additional file 1: Fig. SF1. Using a hashmap, every identified sequence of a sample that is unique, is stored as key and its assigned taxID as value. We call these sequences (keys) as *x-mers*, since they have length $x = k + Y$ (*k*: k-mer length, *Y*: number of consecutive k-mers identified with the same taxID).

Next, all lengths *x* of sequences from the same organism (taxID) are summed up: $A_O = \sum _{i=1}^{n} x_i$, with $x \in X$, the set of *n* distinct x-mers of organism *O*. Only organisms surpassing a threshold of (our default) $A_{OT} = 100$ nucleotides are considered for the next step and final output. This threshold can be adjusted by the user. The higher the threshold, the less organisms are detected. Additional file 1: Fig. SF2 shows how the F1 score can increase with a decrease of detected species in a simulated dataset. Additional file 1: Fig. SF3 shows how much the number of detected species can decrease in an in vitro sample with an increasing threshold. For fair comparisons to the other algorithms, we selected a relatively low threshold of $A_{OT} = 100$ rationally, corresponding to at least one fully mapping read or three independent k-mers.

In order to increase certainty about a specific organism, the abundance measure $A_O$ of every taxID on species level is summed with the abundance of genus and family levels additionally: $A_O = A_S+A_G+A_F$. The intuition behind this is that if an organism is indeed part of the sample, several different areas of it are sampled and processed by chance. This may include areas which are not specific to the organism’s genome. If a species can be detected on species level though, the evidence can be increased by adding higher level counts which have to stem from a specific species in any case. Finally, the organisms can be ranked by their abundance $A_O$. Nevertheless, the full potential unfolds with comparing sample groups with each other.

### Benchmarking Pathonoia

The aim of our algorithm is the reduction of falsely detected organisms in a metagenomic sample and therefore achieving as high precision as possible. Precision can be measured by evaluating a simulated dataset, where the presence (and absence) of every organism is known. Additionally, for testing Pathonoia qualitatively, we evaluated a biological sample (GEO sample *GSM1444167*, HVV-infected fibroblasts [17]), where the ground truth for false-positives can hardly be known. We benchmark Pathonoia against read count-based and Kraken 2-based abundance measuring techniques. We want to emphasize on comparing the technique of measuring abundance and not specific tools. We compare adaptations of Kraken 2 for the aim of specific indication of lowly abundant organisms in a noisy metagenomic sample.

Kraken 2 [11], Centrifuge [12], and Bracken [23] represent the read-count based abundance measures. Kraken 2 and an index of bacterial and viral genomes with minimizer size and k-mer length $l=k=31$ are the baseline for all algorithms in this benchmark. We ran all other abundance measure algorithms (except Centrifuge) on top of the Kraken 2 output, which makes the precision results comparable. No organism can be detected, if it wasn’t detected by Kraken 2 with at least one k-mer (i.e., there is a maximum value for TP and FP). Nevertheless, the way of counting and evaluating species differs in the various algorithms. Our benchmark includes “Kraken 2 with cut-off $\ge 5$ reads”, which means that an organism counts as *detected* when Kraken 2 identifies at least five reads originating from that same organism. The pure Kraken 2 abundance metric counts an organism as *detected*, if at least one read is classified with it. Bracken is another tool based on Kraken which corrects abundance measures, including the statistical distribution of available genomes in the underlying database. Pathonoia may detect organisms with which no read was classified since it evaluates on k-mer level.

For evaluating the performance, we used seven simulated samples, which were constructed by Ye et al. [16] for benchmarking taxonomy classifiers in metagenomics. They are samples containing DNA sequences from twelve to 50 organisms which are found in common human microbiomes or environmental metagenomes, for example the human gut or household.

We oppose the common precision measurement techniques, which indicate if reads were correctly identified or not. Instead, we focus on the (in)correct presence of species (Fig. 2C) and define false positives (FP), true positives (TP) and false negatives (FN) accordingly. Bringing attention to this class, we measure precision (${=\frac{TP}{TP+FP}}$), recall (${=\frac{TP}{TP+FN}}$) and their harmonic mean, the F1 score (${=2\cdot \frac{precision\cdot recall}{precision+recall}}$). Figure 2D shows the average precision, recall and F1 score over all seven samples (detailed values in Additional file 1: Tables ST1 and ST2).

### Downstream analysis

Applying Pathonoia on several samples in a dataset results into a data matrix containing for each sample the abundance measures $A_O$ of all organisms found in at least one of the samples. This abundance data, as well as some metadata about the samples, is the input for the downstream analysis. The goal is to identify differences of pathogenic abundance between sample groups, for example between diseased and control samples. We provide a template analysis script online to which we refer as “guided analysis” Jupyter notebook. In the following we demonstrate the workflow of this analysis exemplary on two datasets (compare Fig. 3).

The datasets used for case study are comprising 48 and 63 samples. The first one is from an in-house study of Frontotemporal Dementia (FTD) [25], looking at brain tissue samples split into disease and control according to Fig. 3A. The second dataset (Fig. 3E) contains liver samples from patients in different fibrotic liver stages due to one of four diseases: Autoimmune Hepatitis (AIH), Non-Alcoholic Fatty Liver Disease (NAFLD), Primary Biliary Cholangitis (PBC) and Primary Sclerosing Cholangitis (PSC).

A Principal Component Analysis (PCA) is executed as a first step of the analysis pipeline. It serves as an “outlier check”, where samples can be identified which may be overly contaminated. Furthermore, the PCA plot is colored by various available metadata (Additional file 1: Fig. SF6) for identifying if a certain experimental setting may result into major contamination. At this step, outlier samples are to be removed from the analysis (manually). In the FTD study we removed 3 samples and in the fibrosis study 12 samples were excluded. (Additional file 1: Fig. SF5) After re-executing the PCA for FTD and coloring according to age, gender, flow cell and RIN score, no bias could be pinpointed based on this metadata.

The group-wise comparison step includes the calculation of the mean abundance per organism for all samples in a group, for example all control samples. DESeq2 [36] is used for this differential abundance analysis. Originally, this tool was developed for differential gene expression analysis. However, it can be used for our purposes since the same statistical model, a Negative Binomial distribution, can be assumed for our data. As argued previously by Simon et al. [8] and Rahman et al. [7] the underlying data source, RNA-seq data, is the same in our case and the gene counts. Metagenomic features, such as our $A_O$, are like gene counts commonly characterized by a high number of zeros and few low count hits. Additional file 1: Fig. SF4 shows this distribution in one of our example datasets.

For increasing the stability of DESeq2, organisms which have zero abundance in most samples are excluded from the analysis. This is usually done for transcriptome analysis as well [37]. The output of this step is a list of organisms alongside their log2 fold change value between the sample groups and a p-value (Wald test) adjusted for multiple testing (with Benjamini-Hochberg). A volcano plot colored by number of samples that an organism is present in, gives an overview of these results. (Results for FTD and fibrosis dataset in Fig. 3 B, F and Additional file 1: Tables ST3 and ST7)

Abundance visualization is the next step. The abundance is plotted per sample group for the most significant organisms. Here, it can be identified, if an organism plays a role in the whole sample group, or if the mean was only elevated due to one extreme case. The latter case should happen less often if more outliers were removed in the first step. In both studies, we observe that most organisms show consistent increase in the diseased samples but not in the controls (Additional file 1: Figs. SF8 and SF10). The outcome of this step is the selection of an *organism of interest*. In the FTD study, we selected *Burkholderia stabilis*, as it was the most prevalent organism of the differentially abundant ones. Its occurrence in the samples is visualized in the PCA plot in Additional file 1: Fig. SF7.

A differential gene expression analysis can help understanding if the organism of interest has a biological origin. Here, the primary transcriptome data is used for comparing samples that contain the organism and samples which do not contain the organism. These sample groups might be subgroup of another sample group. For instance, in the FTD dataset, we only select patient samples for this comparison, to understand which difference *B. stabilis* might make in the diseased case (and since it is not present in any control, also). Using DESeq2 again, on the original read counts, we retrieve a set of up- and down-regulated genes. Ideally, this set concludes as an effect of the presence of the organism of interest. In the FTD study, this step resulted in a set of 143 significantly (p-adj. value $< 0.05$) differentially expressed genes. (More details in Additional file 1: Sect. S3)

We performed a Gene Ontology analysis for understanding the effect of the organism of interest, using an over-representation enrichment analysis (ORA) of the gene sets with WebGestaltR [24]. (For the exact setting, see Additional file 1: Sect. S2.1). For the FTD study, all 34 upregulated genes were compared to the gene sets in the Biological Processes database (Additional file 1: Table ST6).

## Supplementary Information

**Additional file 1.** Supplementary Material.

## Data Availability

A Python package for Pathonoia sample analysis and a guided analysis Jupyter notebook for bulk RNAseq datasets are publicly available on GitHub https://github.com/kepsi/Pathonoia. For benchmarking the algorithm, we used publicly available data on GEO (downloadable through SRA), specifically the datasets with accession numbers GSE61141 and GSE59717 (including highligthed sample GSM1444167). The two case study datasets are the FTD dataset, publicly available at https://ega-archive.org/datasets/EGAD00001008014 and the fibrosis in-house dataset (not publicly available). The intermediate result data for both case studies is available in the given GitHub repository.
