# Supplementary material for: Pathogen detection in RNA-seq data with Pathonoia
Source: BMC Bioinformatics. 2023 Feb 17;24:53. doi: 10.1186/s12859-023-05144-z (PMC9938591; doi:10.1186/s12859-023-05144-z)
Supplement: Supplementary file 1 — Additional file 1. Supplementary Material. [file 12859_2023_5144_MOESM1_ESM.pdf]

SUPPLEMENTARY MATERIAL

Supporting Information - Pathonoia

S1 Pathonoia Algorithm Details

Pathonoia was developed for analyzing the adhering metagenome of an RNA-seq sample or dataset. The algorithm takes FASTA files as input, which are, in the first step, evaluated by the Kraken 2 [1] algorithm. Kraken 2 accepts also fastQ files and single-end, as well as paired-end read files. Pathonoia's code can be easily adjusted to accept those file types as well.

For obtaining the unaligned reads from a host-aligned sample, SAMtools [2] can be used on the BAM file produced by most aligners as follows:

```
samtools aligned.bam view -hb -f 4 > hostUnmapped.bam
samtools view hostUnmapped.bam — awk 'OFS="\t"; print ">" $1 "\n" $10' - > hostUnmapped.fa
```

In case STAR [3] is used for alignment to the host genome, the parameter `--outReadsUnmapped Fastx` can be used for obtaining the unmapped reads.

The Kraken 2 index used for Pathonoia and the results in the main manuscript was built in March 2019 using the following commands:

```
./kraken2-build --download-taxonomy --db db/bacvir_k31
./kraken2-build --download-library bacteria --db db/bacvir_k31 --use-ftp
./kraken2-build --download-library viral --db db/bacvir_k31 --use-ftp
./kraken2-build --build --db db/bacvir_k31 --threads 8 --kmer-len 31 --minimizer-len 31
./kraken2-build --clean --db db/bacvir_k31
```

An updated index from May 2022, created with the same commands, is available for download, on https://oasis.ims.bio/kraken2_k31_2205.zip.

We set the k-mer length equal to the maximal minimizer length in Kraken 2 $k = l = 31$. This forces Kraken 2 to use no optimization (in terms of memory usage) at the cost of precision. You may refer to the original Kraken 2 publication [1] for details. Since the relatively short $k = 31$ (default is $k = 35$) allows for too many random (incorrect) matches to the index, Pathonoia only considers sequences, where at least $z > 4$ k-mers in a row were classified identically (not mentioned in figure). We use $z > 4$ for matching the original k-mer default length of $k = 35$, where our index was built with $k = 31$.

The runtime of Pathonoia is majorly based on Kraken 2, as it takes the majority of runtime and memory of the algorithm. Pathonoia can be considered an "add-on" to Kraken 2 and scans the results in one pass. For example, a dataset of 125 samples ($\sim$ 50GB of fastq.gz files) took 50 min processing time (for the add-on part alone, parallel processing turned off), including the merge of the results, after having run Kraken 2 for all samples within several hours on the same server. Kraken 2's runtime has been benchmarked previously by Ye et al., [5] and is in our experience greatly dependent on the available RAM to load the index, while the actual runtime with 32 threads is around 10 seconds for each sample.

S1.1 Exceptions of discovered species, not discovered by Kraken

Kraken 2 classifies each read with the Lowest Common Ancestor (LCA) of the species corresponding to all k-mers of this read. Pathonoia is based on the same species - k-mer alignments but abstracts from their read

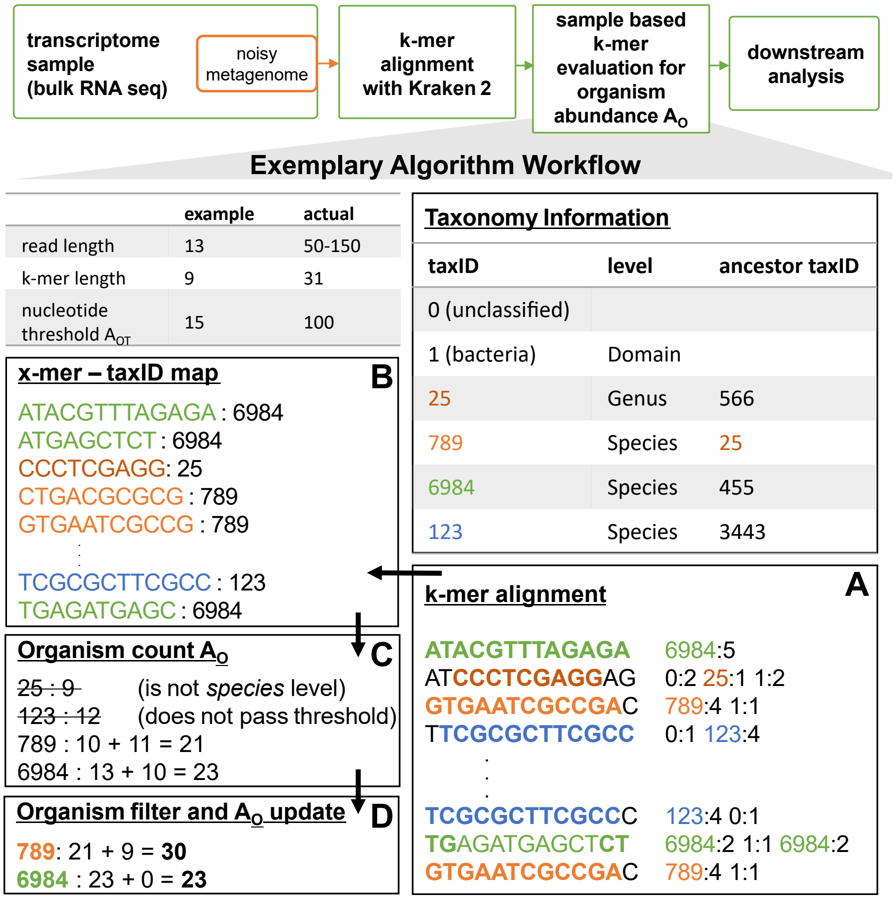

Figure SF1 Pathonoia: Evaluation of all aligned k-mers in a sample. The non-host metagenome of a transcriptomic sample is k-mer-wise aligned to the NCBI nt database of all existing bacterial and viral genomes using Kraken. **A** The aligned k-mers are **B** stored, connected as *x-mers*, in a hashmap with their corresponding taxID. **C** The lengths of x-mers with the same taxID are summed **D** The final abundance A_O is calculated by adding genus and family summed lengths to the species summed length which passes a minimum threshold. (Figure adapted from [4])

"environment". It counts the k-mers of the same species for all k-mers (in all reads) of the same sample. Therefore, it can theoretically be, that some species will not be reported by Kraken 2 that is discovered by Pathonoia. In that case the LCA classification algorithm of Kraken 2 is masking it.

S1.2 Recall, Precision and F1 Score for Benchmarked Algorithms

The following tables contain the underlying data for our benchmark and Figures 2D-E. They are recall, precision and F1 score for the seven simulated samples and their average per evaluated algorithm.

Table ST1 Recall and Precision for simulated dataset comparing Pathonoia with Kraken 2-based algorithms and Centrifuge.

RECALL	Kraken2	K2$\geq$ 5reads	Bracken	KrakenUniq	Centrifuge	Pathonoia
**buccal**	0.750	0.125	0.500	0.583	0.750	0.708
**cityparks**	0.929	0.082	0.480	0.510	0.929	0.673
**gut**	0.756	0.089	0.444	0.467	0.756	0.689
**hous1**	0.785	0.092	0.446	0.462	0.785	0.662
**hous2**	0.865	0.189	0.541	0.541	0.865	0.676
**nycsm**	0.739	0.065	0.413	0.413	0.761	0.609
**soil**	0.931	0.020	0.490	0.510	0.931	0.676
**mean**	0.822	0.095	0.473	0.498	0.825	0.670

PRECISION	Kraken2	K2$\geq$ 5reads	Bracken	KrakenUniq	Centrifuge	Pathonoia
**buccal**	0.044	0.032	0.090	0.090	0.016	0.168
**cityparks**	0.049	0.027	0.137	0.095	0.029	0.274
**gut**	0.029	0.025	0.185	0.057	0.016	0.205
**hous1**	0.056	0.038	0.176	0.111	0.027	0.285
**hous2**	0.033	0.026	0.101	0.066	0.013	0.275
**nycsm**	0.067	0.027	0.157	0.117	0.037	0.226
**soil**	0.036	0.003	0.062	0.063	0.022	0.198
**mean**	0.045	0.025	0.130	0.086	0.023	0.233

Table ST2 F1 Score for simulated samples comparing Pathonoia with Kraken 2-based algorithms and Centrifuge (left). Number of detected species in each sample (right).

F1	Kraken2	K2≥ 5reads	Bracken	KrakenUniq	Centrifuge	Pathonoia
**buccal**	0.084	0.051	0.152	0.156	0.031	0.272
**cityparks**	0.093	0.040	0.214	0.160	0.056	0.389
**gut**	0.056	0.039	0.261	0.102	0.032	0.316
**hous1**	0.105	0.053	0.252	0.179	0.053	0.398
**hous2**	0.063	0.045	0.169	0.117	0.026	0.391
**nycsm**	0.123	0.038	0.228	0.183	0.070	0.329
**soil**	0.068	0.006	0.110	0.113	0.043	0.306
**mean**	0.085	0.039	0.198	0.144	0.044	0.343

DETECTED	Kraken2	K2≥ 5reads	Bracken	KrakenUniq	Centrifuge	Pathonoia
**buccal**	407	94	134	155	1146	101
**cityparks**	1850	298	342	527	3152	241
**gut**	1163	158	108	366	2104	151
**hous1**	907	160	165	270	1868	151
**hous2**	976	274	199	305	2410	91
**nycsm**	509	113	121	162	954	124
**soil**	2674	585	810	821	4359	349
**mean**	1212	240	268	372	2285	173

S1.3 Pathonoia's internal threshold A_{OT}

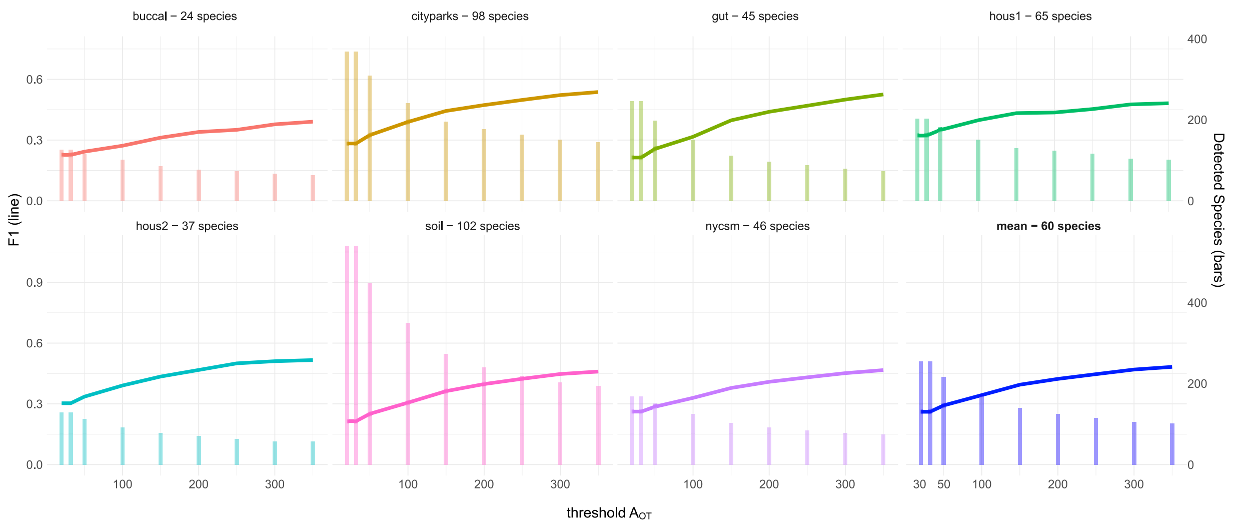

Figure SF2 A_{OT} in simulated dataset: For seven samples and their mean, the number of detected samples with Pathonoia is shown as bars (right y-axis) depending on the chosen threshold (x-axis). The left y-axis and line indicates the improvement of F1 score with increasing threshold. The title of each panel indicates the sample name and number of species considered as groundtruth in it. It is higher than the number of simulated organisms as we consider several strains per organism as detected correctly.

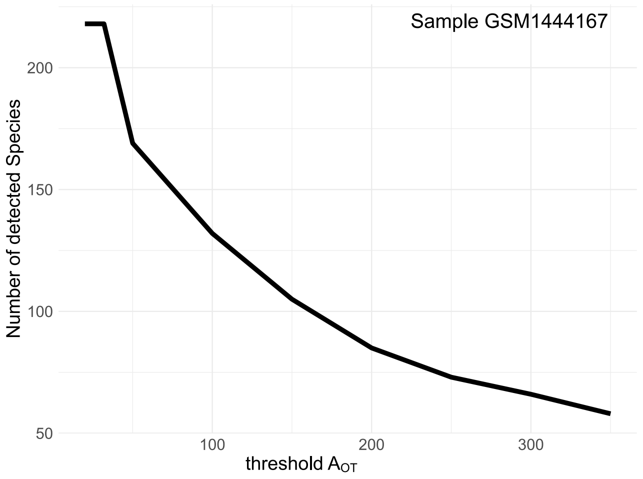

Figure SF3 A_{OT} in Sample GSM1444167: An increase in A_{OT} reduces the number of detected species in examplary *in vitro* sample.

S2 Downstream Analysis

S2.1 Settings for associated tools

For the analysis of our example datasets, we used the STAR aligner [3] for retrieving aligned (and unaligned) reads using the following settings:

```
./STAR-2.5.3a/source/STAR --genomeDir ./indecies/STAR_Human/ --runThreadN 8 --alignIntronMax 0
  --outFilterMismatchNoverLmax 0.06 --outSAMtype BAM SortedByCoordinate
  --outStd BAM_SortedByCoordinate --outSAMunmapped Within --readFilesIn s1.fasta s2.fasta
  --outFileNamePrefix outdir/samplename_STAR_ > outdir/samplename_staralign.bam
```

In the final step of the downstream analysis, we use WebGestalt for the functional enrichment analysis of the (human) gene sets. We use the R version of the tool for the analysis of biological processes and molecular functions, with the following settings:

```
WebGes_BioProc <- function(genes, projectname)
  WebGestaltR(enrichMethod="ORA", organism="hsapiens", enrichDatabase="geneontology_Biological_Process",
  interestGene=genes,interestGeneType="ensembl_gene_id", referenceGeneType="genesymbol",
  referenceSet="genome_protein-coding", minNum=5, maxNum=2000, fdrMethod="BH", sigMethod="fdr", fdrThr=0.05,
  topThr=10, reportNum=20, perNum=1000, nThreads=64, isOutput=TRUE,
  outputDirectory="GO_results/GO_Analysis_biolProcess_FDR05", projectName=projectname, dagColor="continuous",
  hostName="http://www.webgestalt.org/")

WebGes_MolFun <- function(genes, projectname)
  WebGestaltR(enrichMethod="ORA", organism="hsapiens", enrichDatabase="geneontology_Molecular_Function",
  interestGene=genes,interestGeneType="ensembl_gene_id",referenceGeneType="genesymbol",
  referenceSet="genome_protein-coding", minNum=5, maxNum=2000, fdrMethod="BH",sigMethod="fdr",
  fdrThr=0.05,topThr=10, reportNum=20, perNum=1000, nThreads=64, isOutput=TRUE,
  outputDirectory="GO_results/GO_Analysis_molFunction_FDR05", projectName=projectname,
  dagColor="continuous",hostName="http://www.webgestalt.org/")
```

S2.2 Properties of A_O

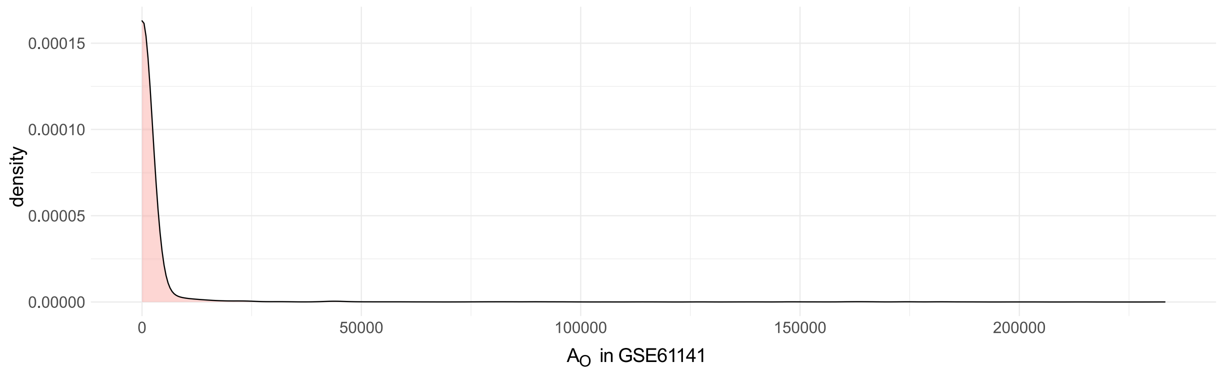

Figure SF4 Negative Binomial distribution of Pathonoia's abundance metric A_O

S3 Supplementary Information for FTD study

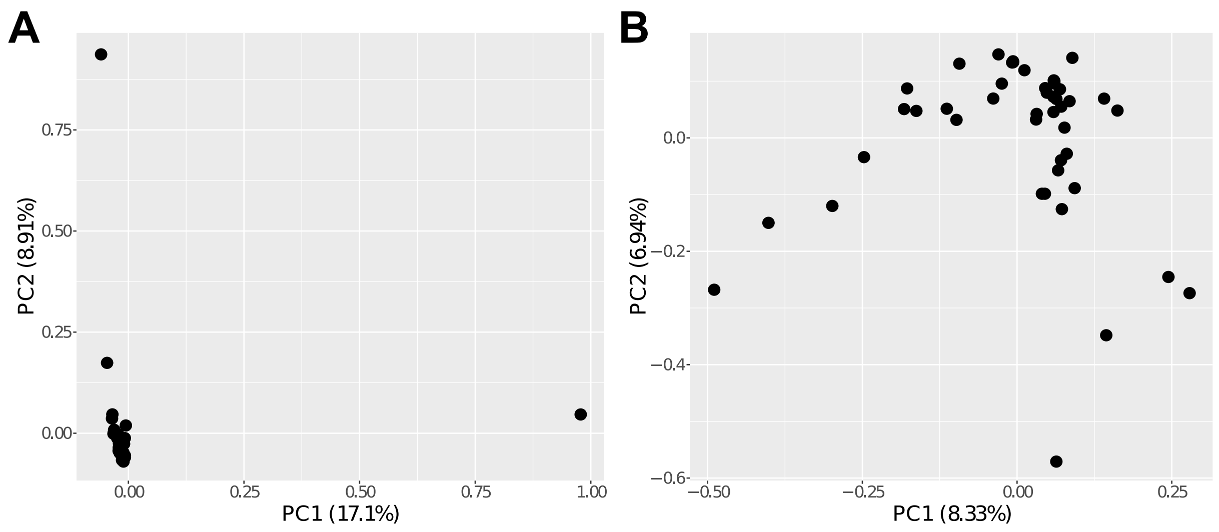

Figure SF5 FTD PCA before (A) and after (B) 5 outlier removal.

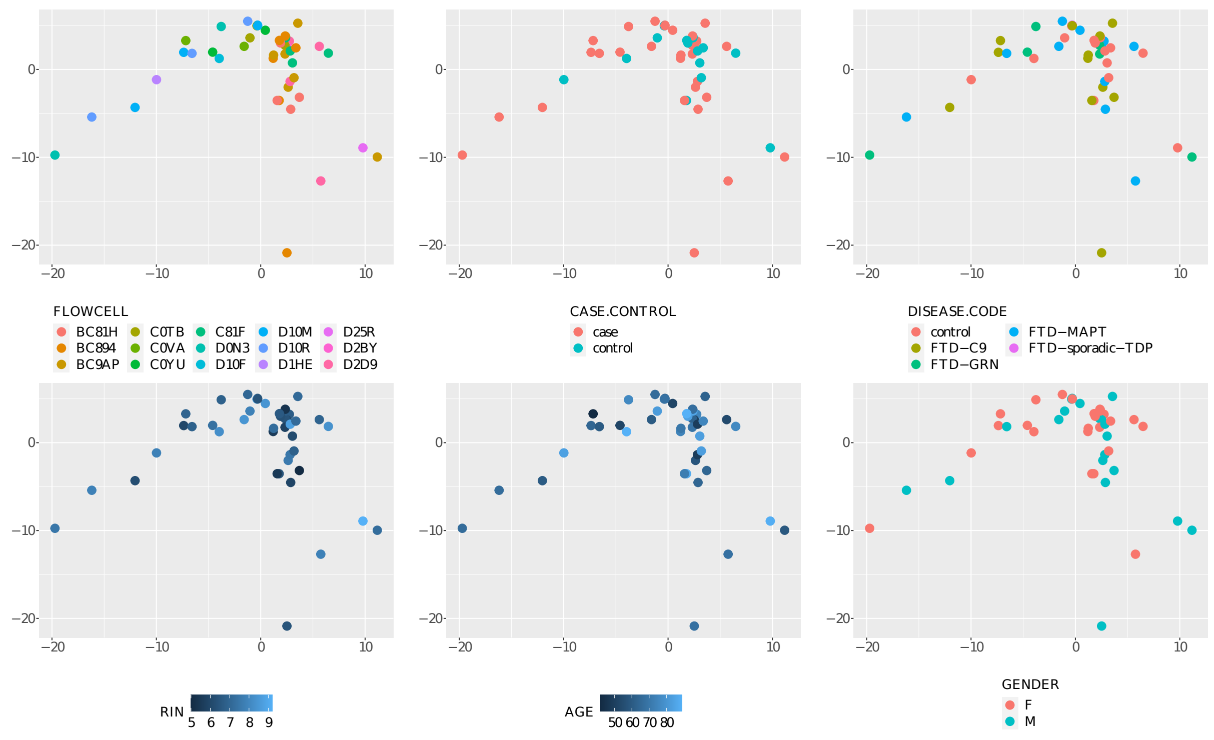

Figure SF6 PCA of FTD dataset colored by different metadata. Clustering cannot be suggested based on the metadata.

Table ST3 All significantly differentially expressed pathogens in brain tissue of FTD patients vs. control

species name	TaxID	base mean	log2FC	padj-value
Flavobacterium indicum	1094466	957.01	-28.88	1.7E-18
**Burkholderia stabilis**	95485	220.14	-26.85	2.5E-18
Caulobacter sp. FWC26	69665	454.84	-27.85	1.2E-17
Paracoccus yeei	147645	152.09	27.49	1.5E-17
Sphingomonas sp. PAMC26645	2565555	131.38	-25.72	2.7E-15
Burkholderia multivorans	87883	181.25	-25.69	2.7E-15
Lactobacillus curvatus	28038	49.67	-24.75	2.9E-14
Neisseriaceae bacterium	2052837	27.47	-24.02	1.6E-13
Staphylococcus hominis	1290	25.26	-23.90	1.9E-13
Arachidicoccus sp. KIS59-12	2341117	8.06	23.61	2.4E-13
Acidovorax sp. 1608163	2478662	17.45	-23.40	5.3E-13
Rheinheimera sp. LHK132	2498451	17.20	-23.38	5.3E-13

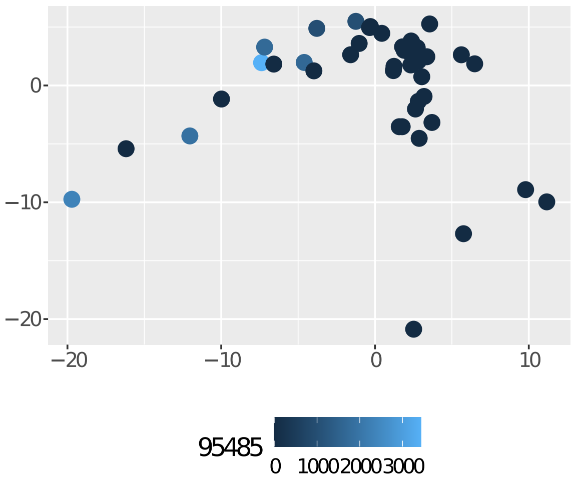

Figure SF7 *B. stabilis* abundance coloring the PCA

Table ST4 Using SEAweb with the search terms "brain" and "*Burkholderia Pseudomallei*" (closest to *B.Stabilis* as the latter was not found in SEA datasets), we find significant differential abundance of *B. Pseudomallei* in two independent datasets concerning neurological diseases comparing patient samples and control.

Entity	Dataset	Condition	Group A	Group B	Covariates	log2 FC	p-adj. value
B. pseudomallei	GSE46131	disease	Lewy body dementia	non-demented	age-gender	-2.29	0.0048
B. pseudomallei	GSE64977	disease	healthy	Huntington's disease	age-gender	1.68	0.0326

Table ST5 List of significantly (p-adj. value < 0.05) up- and down-regulated genes in FTD patient samples with vs. without *B.stabilis* infection.

Upregulated Genes		Downregulated Genes					
MIR570	CFAP47	AC012184.2	SYNPO	ZNF582-AS1	BRSK1	LSM10	TBC1D25
CFAP45	AL645608.1	ACRV1	ELAVL3	SEC16B	TCP11L1	STX1B	DHX30
AC018688.1	AL596247.1	SNORA50B	FAIM2	RUNDC1	KCNA2	POGLUT1	TTC7B
OTOG	GALNT4	AC004706.4	CDHR2	GNB1	PLEKHM3	CD101	VAMP2
AQP4-AS1	RN7SL525P	MRGPRE	ACHE	LINC02192	CAMK1D	MLST8	RHOBTB2
HAUS4	LINC01094	ERAL1	NEFH	CLPTM1	BTBD6	HK1	DMRTC1B
GBP3	CDC20B	EPB41L1	ERICH1	VWA7	PCLO	AC068580.4	PUM2
IL17RE	AC104058.1	SYN1	AES	AL009176.1	KAZN	UBL7	SLC29A1
AL445483.1	KIAA0391	PRDM8	RNF157	CISD3	NOC2LP1	NUDC	SEC16A
RNU2-2P	P2RX4	HAPLN4	TCEA2	AAK1	AL355075.4	ZNF365	SRP68
AL513314.2	SEC1P	AC010970.1	NCKIPSD	ZNF653	AC244517.1	SLC25A44	ASPDH
RF00017	CBR3-AS1	PMS2P6	KCNS1	AC090587.2	FP671120.3	ZNF668	CCNT2-AS1
RGR	PCAT5	CACNG8	CUX2	PSMC5	MAEA	CYP1B1-AS1	AL450992.2
SNORA53	RNF19A	PI4K2A	TMEM39B	SLC9A1	UBE2E2	PHACTR1	OPTN
MIR548H3	IL1RL1	SSU72	LRFN3	CORO2B	BAAT	AKNAD1	SIRPA
RN7SL32P	IL18R1	GSK3A	NEFM	SKIL	LARP1	PRRC2B	
CD24	DOCK7	ATP1A1	SCAMP5	SALRNA1	KCNN1	NOMO1	
PRDM2	HNRNPUL2	KCNC3	DYNLL2	LZTS3	GNG13	HAS1	

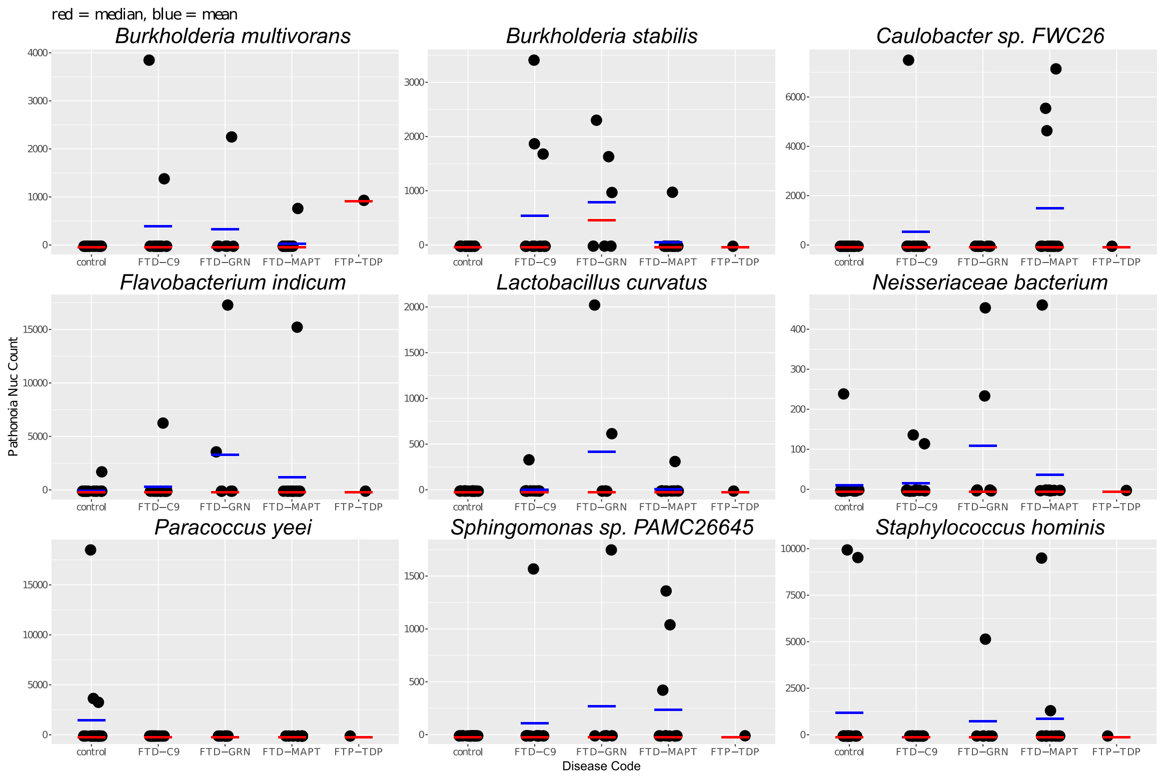

Figure SF8 Top 9 differentially expressed organisms. The Pathonoia abundance for the nine most significant differentially expressed organisms between control and diseased samples is shown. Values are given for all disease phenotypes separately. Mostly the mean (blue bar) and median (red bar) abundance per sample is zero since presence of specific organisms is rare. *Burkholderia stabilis* is the most present organism in seven diseased samples. Even though the analysis was done comparing diseased and control samples, this plot shows the four different phenotypes of FTD. Here it can be seen how big values in single samples can lead to significant group differences. Nevertheless, some of the organisms show consistent increase in the diseased samples but not in the controls.

Table ST6 The top ten biological processes enriched by the set of 34 up-regulated genes in patients with *B.stabilis* present vs patients without this pathogen. The high FDR value for multiple test correction is less informative in this case, as the number of considered genes is low. FDR = 1 for all.

geneSet	description	size	overlap	expect	enrichm.Ratio	pValue
GO:0002825	regulation of T-helper 1 type immune response	27	2	0.021063	94.95157	1.95E-04
GO:0042088	T-helper 1 type immune response	42	2	0.032765	61.04029	4.75E-04
GO:0120163	negative regulation of cold-induced thermogenesis	47	2	0.036666	54.54664	5.95E-04
GO:0071345	cellular response to cytokine stimulus	1015	5	0.791827	6.314513	7.08E-04
GO:0034097	response to cytokine	1100	5	0.858137	5.826573	0.001022
GO:0051241	negative regulation of multicellular organismal process	1143	5	0.891683	5.607376	0.001216
GO:0019221	cytokine-mediated signaling pathway	705	4	0.549988	7.272886	0.001672
GO:0032649	regulation of interferon-gamma production	95	2	0.074112	26.98623	0.002408
GO:0032609	interferon-gamma production	106	2	0.082693	24.18578	0.002987
GO:0032596	protein transport into membrane raft	5	1	0.003901	256.3692	0.003895

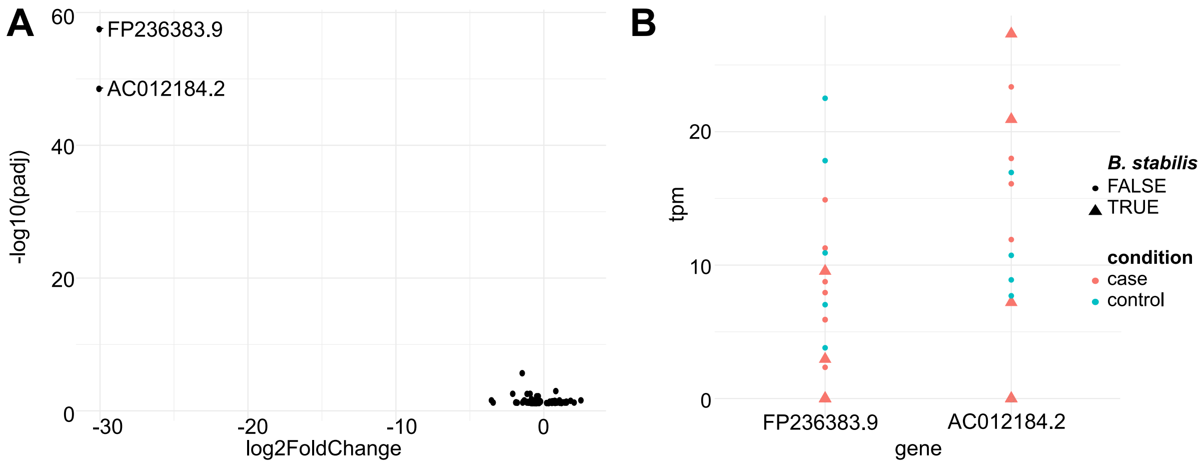

Figure SF9 Diseased samples with and without *B.stabilis* were analyzed for differential gene expression, for understanding how it might be involved in the disease. **A** 143 genes were discovered as significantly differentially expressed with an adjusted p-value < 0.05 (109 down regulated, 34 up regulated). Two down regulated genes show very high log2 fold change combined with an extremely low p-value. These genes are AC012184.2 and FP236383.9. **B** Their expression values (TPM) are given in the individual samples. AC012184.2 is expressed in choroid plexus in the brain according to the Ensemble expression atlas [6]. According to its GeneCard [7] it produces a novel, uncharacterized protein and is involved in ATP binding. For FP236383.9 much less information can be found. It was manually annotated by the Sanger Institute havana project [8], is uncharacterized and to be experimentally confirmed (TEC). According to MirTarBase [9] though, the miRNA *hsa-mir-204-5p* is targeting this gene. Using SEA [10], it can be found that this miRNA is associated with retinal degeneration [11] and that the highest expression of this miRNA is found in eye and brain areas. Furthermore, various brain regions show up in the differential expression analysis results with high significance for this miRNA.

S4 Supplementary Information for Fibrosis study

Table ST7 All significantly (p-adj < 0.05) differentially expressed pathogens in liver tissue of fibrosis patients vs. control (fibrosis level < 1)

tax ID	species name	log2FC	p-adj value	smpls
1858609	*Acidovorax sp. T1*	-29.9827	2.93E-13	10
1114967	*Cutibacterium acnes TypeIA2 P.acn17*	-29.6547	9.04E-11	9
440085	*Methylorubrum extorquens CM4*	-29.3346	1.31E-10	7
28037	*Streptococcus mitis*	-27.8456	3.48E-11	10
1211579	*Pseudomonas putida NBRC 14164*	-27.4035	9.04E-11	10
1636603	*Acinetobacter sp. ACNIH1*	-27.1565	3.14E-09	7
122355	*Pseudomonas psychrophila*	-26.6323	5.95E-09	6
43768	*Corynebacterium matruchotii*	-26.4355	2.93E-13	13
47885	*Pseudomonas oryzihabitans*	-26.3994	7.87E-09	6
1879049	*Acinetobacter sp. WCHAc010034*	-26.2889	8.69E-09	7
401472	*Corynebacterium ureicelerivorans*	-26.0778	1.12E-08	7
739141	*Methylobacterium sp. XJLW*	-25.8098	1.56E-08	6
2219696	*Sphingomonas sp. FARSPH*	-25.4983	2.31E-08	6
1325095	*Bradyrhizobium guangzhouense*	-25.4678	2.31E-08	7
436515	*Variovorax boronicumulans*	-25.2041	3.21E-08	8
2480908	*[Mycobac.] chelonae subsp. gwanakae*	-25.0827	3.64E-08	8
47770	*Lactobacillus crispatus*	-24.8951	4.54E-08	7
120107	*Sphingobium cloacae*	-24.816	4.86E-08	6
1499308	*Paracoccus mutanolyticus*	-24.6895	9.04E-11	11
2014534	*Microbacterium sp. PM5*	-24.6228	6.66E-11	11
71999	*Kocuria palustris*	-24.4215	9.79E-12	12
2082188	*Sphingobium sp. YG1*	-24.2455	1.03E-07	7
1196325	*Pseudomonas putida DOT-T1E*	-24.1474	1.13E-07	4
2282475	*Achromobacter sp. B7*	-24.0824	3.23E-09	9
162426	*Microbacterium hominis*	-23.8223	1.70E-07	7
33010	*Cutibacterium avidum*	-23.6556	2.05E-07	6
945844	*Massilia oculi*	-23.6202	2.07E-07	6
515619	*[Eubacterium] rectale ATCC 33656*	-23.5969	5.00E-10	10
2079596	*Acinetobacter sp. SWBY1*	-23.3826	2.75E-07	5
29466	*Veillonella parvula*	-23.055	4.10E-07	8
216778	*Stenotrophomonas rhizophila*	-23.0221	4.15E-07	5
237610	*Pseudomonas psychrotolerans*	-22.8647	4.94E-07	4
33033	*Parvimonas micra*	-22.5676	7.04E-07	7
1945662	*Paracoccus contaminans*	-22.4732	7.72E-07	4
1123269	*Sphingomonas sanxanigenens ...*	-22.2548	9.91E-07	5
314722	*Pseudoxanthomonas suwonensis*	-21.7936	1.72E-06	5
28131	*Prevotella intermedia*	-21.676	1.92E-06	6
1177574	*Prevotella jejuni*	-21.6626	1.92E-06	3
178339	*Actinomyces hongkongensis*	-21.468	2.38E-06	3
1930557	*Shewanella sp. FDAARGOS_354*	-21.1338	3.48E-06	6
1804984	*Burkholderia sp. OLGA172*	28.86658	1.31E-10	2

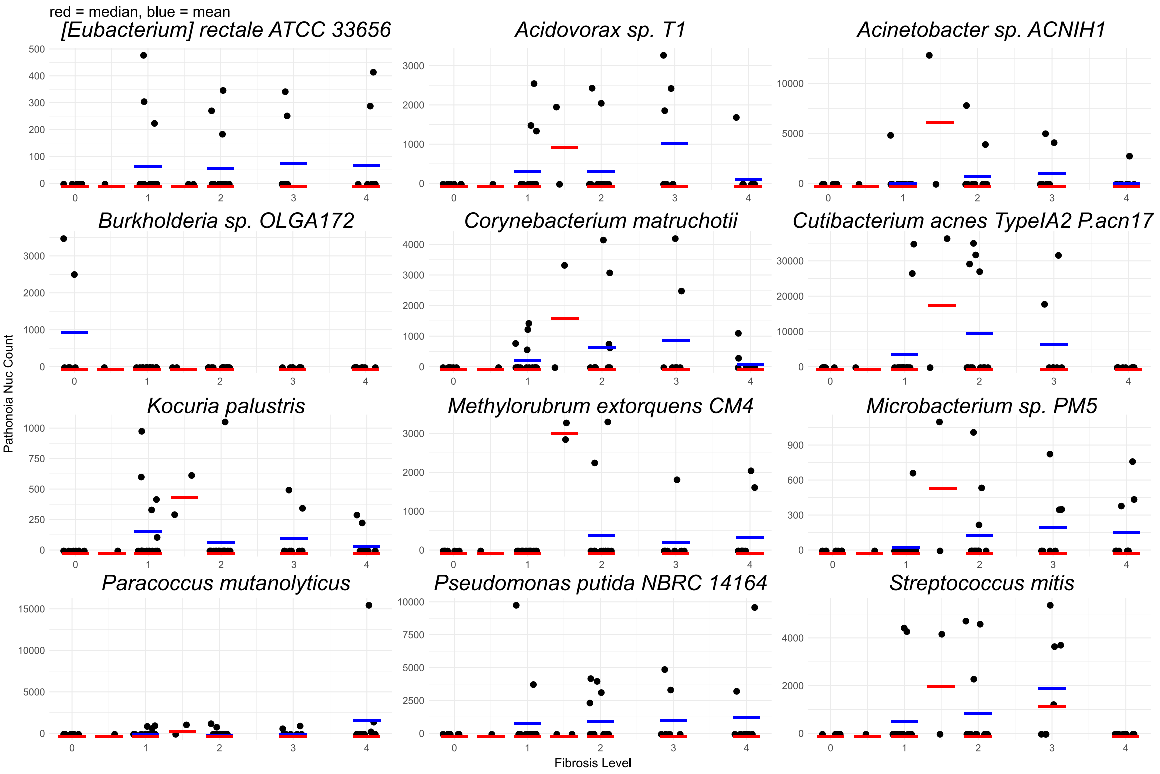

Figure SF10 Top 12 differentially expressed organisms. The Pathonoia abundance for the nine most significant differentially expressed organisms between fibrosis and no fibrosis samples is shown. Values are given for all fibrosis levels separately. Mostly the median (red bar) abundance per sample is zero since presence of specific organisms is rare. Even though the analysis was done comparing fibrosis vs no-fibrosis samples, this plot shows all fibrosis levels. Only *Burkholderia sp. OLGA172* shows abundance in non-fibrotic samples, but only two samples contain this pathogen.

References

1. Wood, D.E., Lu, J., Langmead, B.: Improved metagenomic analysis with kraken 2. Genome biology **20**(1), 1–13 (2019)
2. Danecek, P., Bonfield, J.K., Liddle, J., Marshall, J., Ohan, V., Pollard, M.O., Whitwham, A., Keane, T., McCarthy, S.A., Davies, R.M., Li, H.: Twelve years of SAMtools and BCFtools. GigaScience **10**(2) (2021)
3. Dobin, A., Davis, C.A., Schlesinger, F., Drenkow, J., Zaleski, C., Jha, S., Batut, P., Chaisson, M., Gingeras, T.R.: STAR: ultrafast universal RNA-seq aligner. Bioinformatics **29**(1), 15–21 (2012)
4. Liebhoff, A.-M.: Detection of Pathogenic Infections in Neurological Disorders Through Recycling of Gene Expression Data. Cuvillier Verlag, Göttingen (2021)
5. Ye, S.H., Siddle, K.J., Park, D.J., Sabeti, P.C.: Benchmarking metagenomics tools for taxonomic classification. Cell **178**(4), 779–794 (2019)
6. Papatheodorou, I., Moreno, P., Manning, J., Fuentes, A.M.-P., George, N., Fexova, S., Fonseca, N.A., Füllgrabe, A., Green, M., Huang, N., *et al.*: Expression atlas update: from tissues to single cells. Nucleic acids research **48**(D1), 77–83 (2020)
7. Stelzer, G., Rosen, N., Plaschkes, I., Zimmerman, S., Twik, M., Fishilevich, S., Stein, T.I., Nudel, R., Lieder, I., Mazor, Y., *et al.*: The genecards suite: from gene data mining to disease genome sequence analyses. Current protocols in bioinformatics **54**(1), 1–30 (2016)
8. Sanger Institute: Manual Annotation - Havana. (2020). https://www.sanger.ac.uk/project/manual-annotation (accessed August 5, 2020)
9. Hsu, S.-D., Lin, F.-M., Wu, W.-Y., Liang, C., Huang, W.-C., Chan, W.-L., Tsai, W.-T., Chen, G.-Z., Lee, C.-J., Chiu, C.-M., *et al.*: mirtarbase: a database curates experimentally validated microrna–target interactions. Nucleic acids research **39**(suppl_1), 163–169 (2011)
10. Rahman, R.-U., Liebhoff, A.-M., Bansal, V., Fiosins, M., Rajput, A., Sattar, A., Magruder, D.S., Madan, S., Sun, T., Gautam, A., *et al.*: Seaweb: the small rna expression atlas web application. Nucleic acids research **48**(D1), 204–219 (2020)
11. Arora, A., Guduric-Fuchs, J., Harwood, L., Dellett, M., Cogliati, T., Simpson, D.A.: Prediction of micrornas affecting mrna expression during retinal development. BMC developmental biology **10**(1), 1–15 (2010)
